# Simulation of Population-Based Commuter Exposure to NO_2_ Using Different Air Pollution Models

**DOI:** 10.3390/ijerph110505049

**Published:** 2014-05-12

**Authors:** Martina S. Ragettli, Ming-Yi Tsai, Charlotte Braun-Fahrländer, Audrey de Nazelle, Christian Schindler, Alex Ineichen, Regina E. Ducret-Stich, Laura Perez, Nicole Probst-Hensch, Nino Künzli, Harish C. Phuleria

**Affiliations:** 1 Swiss Tropical and Public Health Institute (Swiss TPH), Socinstrasse 57, P.O. Box, Basel 4002, Switzerland; E-Mails: m.tsai@unibas.ch (M.-Y.T.); c.braun@unibas.ch (C.B.-F.); christian.schindler@unibas.ch (C.S.); alex.ineichen@unibas.ch (A.I.); regina.ducret@unibas.ch (R.E.D.-S.); l.perez@unibas.ch (L.P.); nicole.probst@unibas.ch (N.P.-H.); nino.kuenzli@unibas.ch (N.K.); 2 University of Basel, Petersplatz 1, Basel 4003, Switzerland; 3 Department of Environmental and Occupational Health Sciences, University of Washington, Seattle, WA 98195, USA; 4 Centre for Environmental Policy, South Kensington Campus, Imperial College London, London SW7 2AZ, UK; E-Mail: anazelle@imperial.ac.uk; 5 Centre for Environmental Science and Engineering, Indian Institute of Technology, Powai, Bombay, Mumbai 400076, India; E-Mail: phuleria@iitb.ac.in

**Keywords:** air pollution, model comparison, traffic, travel mode, travel pattern

## Abstract

We simulated commuter routes and long-term exposure to traffic-related air pollution during commute in a representative population sample in Basel (Switzerland), and evaluated three air pollution models with different spatial resolution for estimating commute exposures to nitrogen dioxide (NO_2_) as a marker of long-term exposure to traffic-related air pollution. Our approach includes spatially and temporally resolved data on actual commuter routes, travel modes and three air pollution models. Annual mean NO_2_ commuter exposures were similar between models. However, we found more within-city and within-subject variability in annual mean (±SD) NO_2_ commuter exposure with a high resolution dispersion model (40 ± 7 µg m^−3^, range: 21–61) than with a dispersion model with a lower resolution (39 ± 5 µg m^−3^; range: 24–51), and a land use regression model (41 ± 5 µg m^−3^; range: 24–54). Highest median cumulative exposures were calculated along motorized transport and bicycle routes, and the lowest for walking. For estimating commuter exposure within a city and being interested also in small-scale variability between roads, a model with a high resolution is recommended. For larger scale epidemiological health assessment studies, models with a coarser spatial resolution are likely sufficient, especially when study areas include suburban and rural areas.

## 1. Introduction

Daily travel within urban areas is an important component of human exposure to traffic-related air pollutants. Levels of traffic-related air pollutants such as nitrogen dioxide (NO_2_), ultrafine particles (UFP), and carbon monoxide (CO) have consistently been shown to be higher in urban areas and in transit-related environments than at other non-occupational locations [1,2,3,4]. In Europe, people spend about 8% of the day in transport environments [5]. Many of those daily trips, especially the commutes to and from work or school, generally take place during times of the day with peak traffic flow and thus high concentration levels. In most epidemiological studies on health effects of long-term exposure to traffic-related air pollution, however, in-transit exposure is ignored [6]. The exposure assessment of these studies mostly relies on estimated levels at no more than one fixed-site per person such as average level of the respective pollutant at the person’s home or a fixed monitoring station nearby, or on traffic indicator variables, including distance to major roads and traffic intensity [6]. With technological advances and the development of personal monitors, several personal monitoring studies have been carried out to better quantify air pollution exposures in traffic (e.g., [1,7,8,9,10,11]). Although personal exposure studies provide important insights into exposure determinants, such studies are generally not feasible for large cohort studies due to the high costs and the commitment required of the study participants.

Only a few modeling approaches exist to estimate air pollution exposure in transit. Some long-term exposure assessment studies have applied the concept of microenvironments to take into account in-transit exposure (so-called compartment models). This approach uses the average concentrations within different transport environments, derived from personal or fixed station measurements, and multiplies them by the time spent in such microenvironments. While some studies differentiate between several transportation modes [12], others use only a general “transport” microenvironment [5]. For both approaches, uncertainties remain for pollutants with high spatial and temporal variability within microenvironments, such as for example NO_2_ concentrations, thus creating inter-subject variability [13,14]. More dynamic models account for people’s specific location throughout the day along with time-activity information and in-transit patterns. Exposures are estimated by overlaying air pollution models with information from census data, time-activity and/or geo-coded origin-destination information from surveys [13,15,16,17]. Another approach integrates activity-based transport models simulating spatially and temporally resolved vehicle volume, traffic emissions, and population density to predict population exposure [18,19,20]. Limitations of such exposure simulations include the imprecision of spatial in-transit data. Some models simulate trips as straight lines or the shortest or fastest route along roads between locations and zones without knowing the exact route and/or travel mode. Others are based on a synthetic population and routes are generated stochastically as in the case of activity-based transport models.

Simulated in-transit exposure estimates might be further impaired by the limited spatial resolution of the air pollutant models used which do not accurately represent the high spatial variability of pollutant concentrations, especially in urban streets [18,21]. Developing models of high resolution requires expertise, adequate data and can be costly. Air pollution models commonly used to assess long-term exposure to traffic-related NO_2_ include inverse-distance weighted interpolation of monitoring data (e.g., [15]), dispersion models (e.g., [17,22]) and land use regression (LUR) models (e.g., [23,24]). While dispersion models mostly rely on dispersion theory, emission and meteorological data, LUR models apply regression techniques using actual air pollution monitoring data and predictor variables obtained from geographic information systems (GIS). More recently, hybrid models were also developed (e.g., [25]) which combine personal or regional monitoring with other air pollution modeling methods [26].

Better quantification of daily in-traffic exposure of a general population is important to provide better estimates of total air pollution exposures in investigations of long-term health effects. The aim of this study was to develop an approach to estimate individual NO_2_ exposures in a representative sample of the population during commute within the metropolitan area of Basel (Switzerland). Our approach includes spatially and temporally resolved data on commuter trips within the study area, and three annual air pollution models with varying spatial resolution. This paper describes the simulation of commuter routes and the in-transit NO_2_ exposure from outdoor origin. It also evaluates the differences between these NO_2_ commuter estimates for the three models that may occur when applying them in long-term exposure assessments. The potential bias that can occur when ignoring these commute exposures but rely on home outdoor locations only in epidemiological studies on the long-term health effects of traffic-related outdoor air pollution is explored in Ragettli *et al.* [27]. As in many epidemiological studies, we chose NO_2_ as marker for traffic-related outdoor air pollution as it describes the spatial distribution of traffic-related air pollution well. But, in principle, the simulation is applicable to any other traffic-related pollutant.

## 2. Methods

### 2.1. Study Area

**Figure 1:**
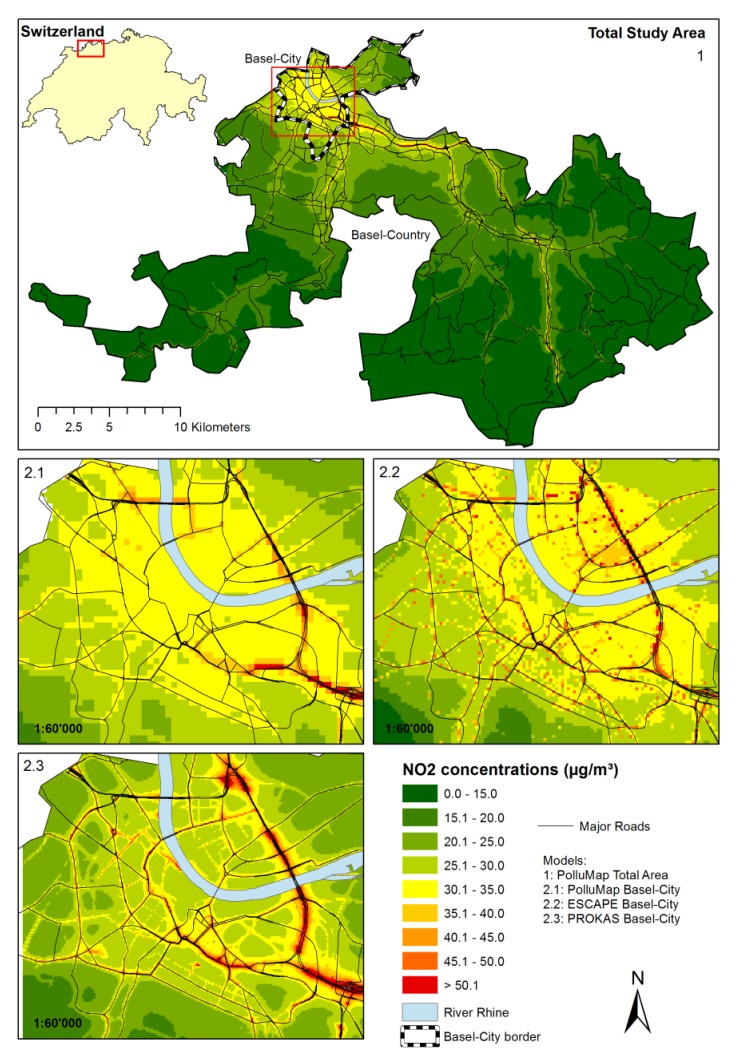
Annual mean NO_2_ concentrations from different air pollution models for total study area (**1**); and Basel-City (**2**).

Our study was carried out in the region of Basel (Switzerland), which covers the two Swiss Counties (called Cantons) of Basel-City and Basel-Country (Figure 1). The area (550 km^2^) includes a population of 465,000 people. While the Canton of Basel-City is a predominantly urban area with buildings of usually three to five stories, Basel-Country is largely suburban and rural in character. Hereafter, we differentiate Basel-City from the total study area and present results separately. The region is a relatively low-pollution area with an annual mean NO_2_ suburban background concentration of 23.5 µg m^−3^ in 2010.

#### 2.1.1. Study Population and Commuter Routes

**Figure 2:**
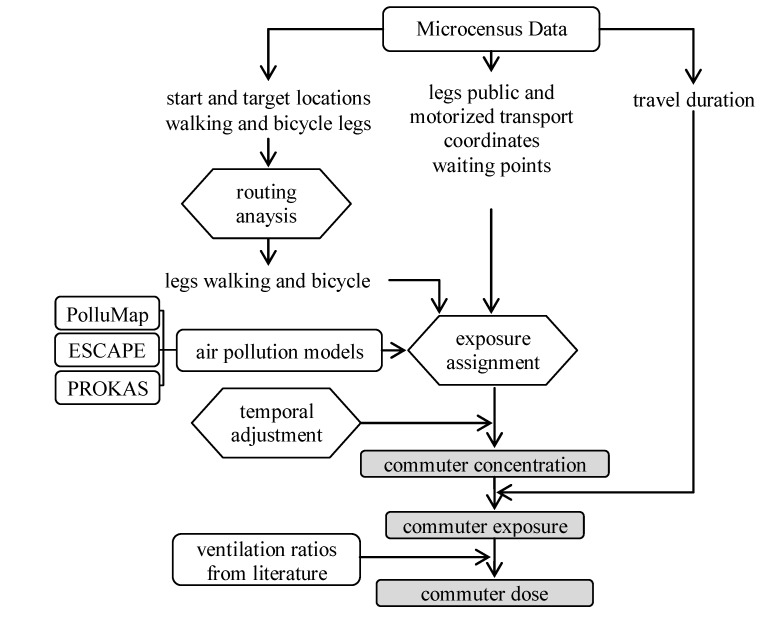
Schematic representation of the applied methodology. Boxes are inputs, and hexagons are analysis steps. Shadowed boxes indicate commuter estimates.

The study methodology is illustrated in Figure 2. We extracted information on commuter routes from the year 2010 Swiss Mobility and Transport Microcensus [28]. Our focus was on commutes between home, work or school locations as those trips account for a large fraction of travel time on work days and are usually carried out regularly over time. The telephone-based survey included geo-coded time-activity diaries covering one day of a representative number of randomly selected individuals of each Swiss Canton. Geo-codes were recorded for start locations, trip destinations and places where study participants changed their mode of transport during trips. In addition, the actual route of public transport and motorized transport legs were simulated based on the coordinates by an interactive routing tool during the interview. A leg is defined as each contiguous part of a trip that is covered with the same travel mode. For example, a trip of a person who walks to the train station, takes the train and then walks to work from the destination train station covers three legs. The routing was performed based on the TeleAtlas MultiNet road network and a public transport network with integrated time table. All public transport and motorized transport routes ≥3 km were verified during the interview.

We subsequently simulated both walking and bicycle routes based on the geo-coded start and target locations using the GIS based route finder Network Analyst by ESRI (ArcGIS 9.3, Redland, CA, USA). The routing was performed using the Swiss GIS road network VECTOR25 (Federal Office of Topography swisstopo, Wabern, Switzerland, 2008) which has been shown to be more complete than the TeleAltas road network for smaller side streets and pedestrian roads [29]. The shortest routes between the geo-codes were determined using the distance, *i.e.*, the road segment length, as the cost factor in the analysis. We validated the routing performance of the GIS model by comparing real commutes of test persons with the simulated routes (for details, see Supplementary Section 1). Additionally, a quality check of the simulated distance *versus* reported distance of the Microcensus data of all legs and travel modes assured that large detours were avoided. Comparisons between the reported distance and routing distance of walking and bicycle legs showed moderate to high agreement (*R*^2^ = 0.6 to 0.8) for both the microcensus data and validation study (see Supplementary Section 2).

To evaluate the benefit of verifying car legs ≥3 km instead of modeling the shortest routes in terms of driving time between origin and destination location, we also simulated the fastest route based on the TeleAtlas road network for subjects who travel only by car between home and work/school locations (results of this sub-analysis are provided in the Supplementary Section 3).

We classified each commuter leg as either a main or a side street based on the longest road segments of the underlying road network. Major streets in TeleAtlas were defined as the functional road classes (FRC) 0–4. In VECTOR25, streets classified as highways and class 1 roads were used as main roads. Public transport legs that were not directly overlapping with the TeleAtlas road network were classified based on the length of the nearest road segments within a buffer of 15 m.

A total of 736 subjects (28% of all respondents with time-activity information in the study area) were selected based on the following inclusion criteria: (a) living and working, or attending a school within the study area, locations not being the same place; (b) reporting at least one trip between home and work location within the study area classified by purpose working or education; (c) quality of geo-codes being of sufficient quality (house number or street level). Indirect commuter trips, including for example a stop at a shop or day care, were also included. If an uneven number of trips between home and work/school location were reported (11% of the total), *i.e.*, a trip from either home to work or vice-versa was missing, the reported single trip was duplicated. For these cases the time of the day when leaving home, work or school was used as time information.

### 2.2. Air Pollution Models

We used three spatially resolved annual mean ambient air pollution models to estimate exposure to NO_2_ during commute. The models were all originally developed for estimating outdoor air pollution exposure at home outdoor locations. Two models for Basel-City and one for the total study area were available (see Table 1). The first model, PROKAS, was developed for the calculation of traffic induced air pollution for the Basel department of air hygiene. It consist of a Gaussian plume model (PROKAS_V) to estimate the urban traffic background concentration for a given road network and meteorology, and an integrated building structure module (PROKAS_B) [30]. The latter is used to account for the rather complex built environment of urban areas. It is based on pre-calculated dimensionless concentrations for 20 different building structures and 36 air flow directions determined by the microscale dispersion model MISKAM [31]. Additional NO_2_ concentrations such as household (heating), shipping traffic from the river Rhine, industry and commerce were estimated with the three-dimensional model LASAT [32] and overlaid with the traffic-related NO_2_ concentrations. The road transport emissions for all major roads were computed by a local traffic model (mobility department Basel-City) and projected to the TeleAtlas road network. The second model, ESCAPE, was developed within the framework of the European Study of Cohorts for Air Pollution Effects (ESCAPE) using LUR modeling based on 2009 NO_2_ measurement data at 40 locations [33]. Given that the model was designed to estimate NO_2_ exposures at home outdoor locations and not in transport environments *per se*, we applied the LUR model to a 50 × 50 m grid corresponding to the quality of the model input data. More information on the Basel ESCAPE model is provided in the Supplementary Section 4. Finally, a nationwide dispersion model, PolluMap, was available from the Swiss Federal Office for the Environment (FOEN). The nationwide model computes source-specific annual concentrations based on a Gaussian plume model using emission inventories from 2010, a national road network map and meteorological data. Emission inventories considered include road traffic, rail traffic, aviation, industry, commerce, construction, household (heating), agriculture and forestry [34].

**Table 1:** Characteristics of the three air pollution models used to individually assign commute exposure.

	Models		
	PROKAS	ESCAPE	PolluMap
Year	2010	2009	2010
Grid size	25 × 25 m	50 × 50 m	100 × 100 m
Method	Gaussian dispersion, integrated building characteristics	Land use regression	Gaussian dispersion
Availability	Basel-City	Basel-City	Switzerland
Comparison with measurements	NA	*R*^2^ = 0.67 ^a^	*R*^2^ = 0.80 ^b^
Reference	Air Hygiene Department Basel and Lohmeyer 2008 [30]	Beelen *et al.* 2013 [33]	Federal Office for the Environment Switzerland (FOEN) [34]

Note: ^a^ unadjusted *R*^2^; ^b^ Measured values are the arithmetic mean of the three annual averages 2008, 2009, 2010.

### 2.3. NO_2_ Exposure Assessment

We overlaid maps (Figure 1) of annually averaged ambient NO_2_ concentrations from the three air pollution models on the commuter legs to estimate commuter exposure. NO_2_ concentration of a leg (C_leg_) was computed based on the sum of the extracted NO_2_ grid concentrations (C_grid_) weighted by the length of the leg within the grid (Equation (1)):

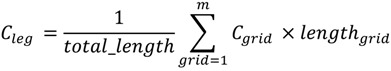
(1)


We calculated temporal adjustment factors for each hour of the day separately for main roads and side streets to consider the diurnal pattern of NO_2_ levels and road-type specific differences in hourly traffic volume and composition of vehicles. NO_2_ data (30-min averages) from two fixed air pollution monitoring stations, a street site and an urban background site within the Canton of Basel-City, were used to derive the ratios. Ratios were computed between the annual weekday hourly means and the annual mean concentration measured at the monitoring stations for main streets (*ratio_m-h_*) and side streets (*ratio_s-h_*) (for more details on ratios, street class distribution by travel mode see Supplementary Section 5). We then applied ratios to each leg concentration *C_leg_* based on the road classification and start hour of the leg to compute subjects’ commuter NO_2_ concentration, *C_subject_* (Equation (2)) and exposure, *E_subject_* (Equation (3)). For the calculation of subjects’ commute exposure, waiting time between two legs (e.g., when transferring from one mode to another for example at public transport stops) and respective NO_2_ concentrations (C_wait_) were also considered:

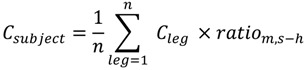
(2)

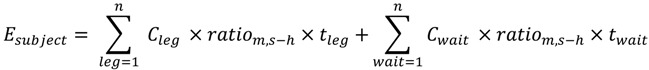
(3)
where t_leg_ is the duration spent on the leg, and t_wait_ the time spent at a waiting location. Time-weighted commuter exposure is defined as the exposure divided by the total commuter duration of a subject. We used the reported travel time and waiting time information from the microcensus data for all travel modes.

Finally, as a proxy for the inhaled dose, we derived adjusted estimates of exposure, taking into account mode-specific ventilation rates (we use the term “dose” hereafter). Since neither physical activity measures nor adequate data on body weight and body height were available, we applied ventilation ratios extracted from the literature to each leg. A ratio of 1.7 [8] for walking and 2.0 for bicycle [8,35], respectively, relative to public transport and motorized transport was assumed.

Comparisons between the three air pollution models based on subjects’ commuter NO_2_ estimates (*i.e.*, concentration, exposure and dose) and by travel mode (*i.e.*, legs without waiting time) were then performed to evaluate the potential differences in outdoor NO_2_ estimates that may arise when applying models with varying modeling techniques, spatial resolution and input data. A validation of the in-transit NO_2_ exposure estimates—for the overall population and by travel mode—was neither the purpose of this study nor possible due to the unavailability of reliable real-time personal NO_2_ monitoring devices with appropriate sensitivity and specificity. As our focus was the long-term exposure to outdoor air pollution in transport environments, the benefit of validating the annual models with short-term personal measurements is limited. However, to evaluate the performance of the air pollution models, we compared the PROKAS, ESCAPE and PolluMap model to NO_2_ measurements from a total of 31 monitoring sites within Basel-City from the Swiss study on Air Pollution and Lung and Heart Diseases in Adults (SAPALDIA) (see Supplementary Section 6). These measurements were conducted outside subjects’ homes in three biweekly integrated sampling campaigns in 2011 using Passam passive diffusion samplers (Passam AG, Schellenstrasse, Männedorf, Switzerland). We compared the average ambient NO_2_ concentrations of each site to the respective grid value of the three models. The data analyses were conducted using the statistical software STATA (version 12.1, STATA Corp., College Station, TX, USA).

## 3. Results

### 3.1. Commuter Behavior of the Study Population

The majority (84%) of the study population reported two commuter trips per day. The remaining population traveled four times per day between home and work/school locations. The average number of legs (±standard deviation (SD)) per subject and day in Basel-City and the total area was 4.6 (±3.0) and 4.6 (±2.9), respectively. A summary of the characteristics of the study population (age, sex, working hours per week) is shown in Table S5 in the supplement. In the total study area, the main travel modes used for the daily commute to work/school (defined as the mode used for the longest distance of the commute trips per day) were motorized transport (car and motorcycle; 32%) and public transport (bus, tram, train; 30%). However, within Basel-City, the active transport (walking: 27%, bicycle: 30%) was the main travel mode, followed by public transport (32%). Motorized transport was used by 9% of the subjects living and working in Basel-City.

The average daily commuting distance within Basel-City was about half of that of the total study area (Table 2). However, the average trip duration between home and work/school locations (18.2 ± 11.5 min in Basel city) was only 14% shorter. Average daily travel time for all main travel modes were rather similar within Basel-City (30–35 min), except for public transport, which was about twice as long (62 min). Commuting mainly took place within the rush hours 6–8 am and 4–6 pm (Figure S4), coinciding with the diurnal peaks of air pollution.

**Table 2:** Daily commuter distance and commuter duration of subjects per main travel mode and study area.

	Basel-City					Total Area				
	n (subjects)	mean	(sd)	min	max	n (subjects)	mean	(sd)	min	max
**commute distance (in m)**										
all modes	258	6,086	(4,588)	52	29,095	736	13,976	(15,329)	23	88,346
walking	69	2,965	(2,239)	328	16,126	140	2,480	(2,043)	23	16,126
bicycle	78	5,325	(3,583)	52	26,426	131	5,627	(3,910)	52	26,426
motorized transport	22	9,128	(4,128)	3,569	17,136	234	21,318	(17,610)	877	88,346
public transport	83	8,801	(5,082)	3,261	29,095	219	19,081	(15,204)	2033	83,182
other	6	3,153	(1,981)	1,316	6,310	12	2,882	(2,259)	1061	7,895
**Commute duration (in minutes)**										
all modes	258	42	(25)	4	155	736	49	(33)	2	204
walking	69	35	(24)	9	155	140	32	(25)	2	155
bicycle	78	30	(15)	4	90	131	32	(19)	4	125
motorized transport	22	35	(14)	19	64	234	43	(26)	4	163
public transport	83	62	(24)	23	140	219	78	(32)	23	204
other	6	32	(17)	20	63	12	31	(20)	6	74

Note: sd: standard deviation; min: minimum; max: maximum.

### 3.2. Comparison of Air Pollution Models

In the overall comparison between the model-based NO_2_ estimates and the SAPALDIA NO_2_ measurements, the PROKAS model obtained best agreement (*R*^2^ = 0.58) whereas correlations were lower but similar for the ESCAPE (*R*^2^ = 0.41) and the PolluMap model (*R*^2^ = 0.46). While the PROKAS model predicted the street sites concentrations better than the other models, the urban background sites showed good agreement also with the nation-wide dispersion model PolluMap, which had the lowest resolution (see Supplementary Section 6).

Summary statistics of estimated time-weighted subjects’ commuter NO_2_ exposure during commute using the three air pollution models are shown in Table 3. Within Basel-City, mean and median NO_2_ concentrations and exposures were similar between the models. However, as illustrated by the standard deviations and confirmed by the Fisher Pitman test, the variability and range of model estimates were significantly increased with higher model resolution. Covering the total study area, the PolluMap model also allowed comparisons of within Basel-City commuter exposures to commutes within the total study area, *i.e.*, including subjects traveling between the two Cantons and within Basel-Country. Average exposure estimates from the PolluMap model for the total study area were ~5 µg m^−3^ lower than within Basel-City, and the range was twice as large because of the smaller values on the lower end.

**Table 3:** Summary of time-weighted subjects’ NO_2_ exposure during commute (in µg m^−^³) for Basel-City by air pollution model, and for the total area (only one model available).

	Model	n (subjects)	mean	(sd)	min	p5	median	p95	max
Basel-City	PROKAS	258	39.9	(6.5)	20.7	29.3	40.1	49.7	61.4
	ESCAPE	258	40.8	(5.4)	23.8	31.8	41.3	49.7	53.8
	PolluMap	258	38.8	(4.7)	24.1	30.3	39.2	46.0	51.0
Total area	PolluMap	736	33.7	(7.7)	12.4	19.8	34.8	45.0	52.2

Note: sd: standard deviation; min: minimum; p5: 5th percentile; p95: 95th percentile; max: maximum.

In general, both leg and subject specific NO_2_ concentrations correlated well between the models (*r* = 0.81–0.91, Table S6). NO_2_ concentrations from PROKAS showed higher correlations with ESCAPE (the second highest resolution model) than with PolluMap, the lowest resolution model. Spearman correlation coefficients of subjects’ NO_2_ commuter exposures and dose estimates were almost identical for all model pairs and were close to 1.0 (Table S6).

**Figure 3:**
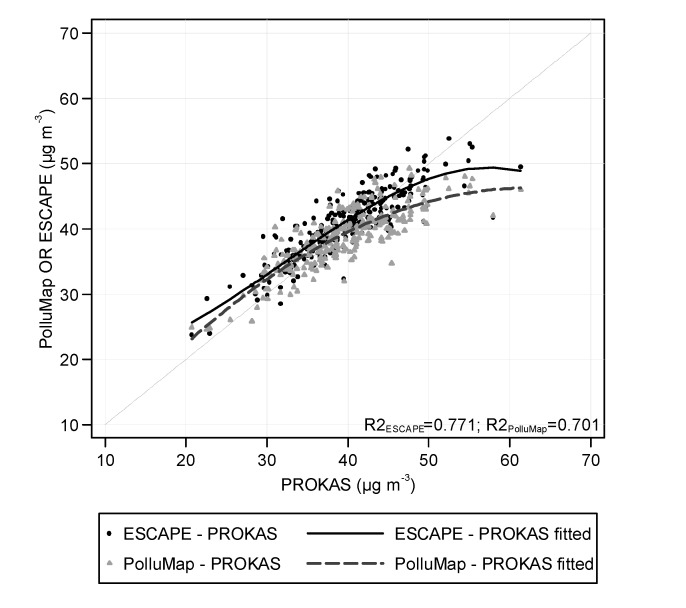
Scatter plot comparing subjects’ estimated commuter NO_2_ concentration based on the high spatial resolution model (PROKAS) with the estimates from PolluMap and ESCAPE models, respectively, using subjects from Basel-City (*n* = 258).

**Figure 4:**
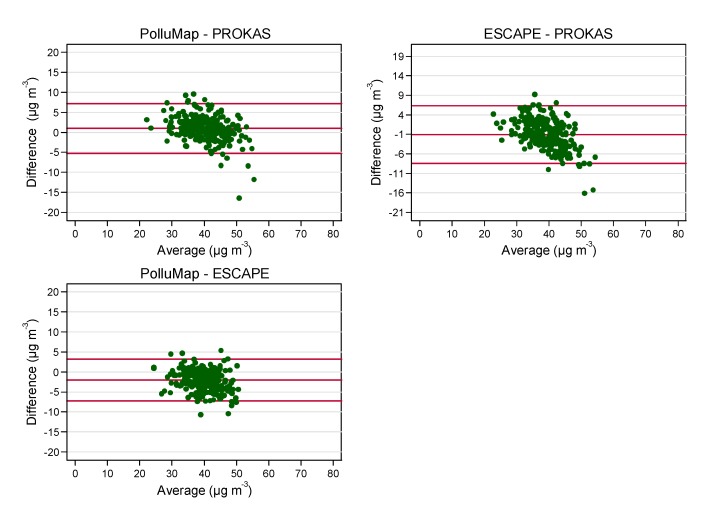
Bland Altman plots of time-weighted commuter NO_2_ exposure of subjects commuting within Basel-City (*n* = 258). The lines represent the mean difference ±2 × standard deviation.

As illustrated in Figure 3 and Figure 4, we observed a non-linear relationship between model estimates. Compared to PROKAS, a systematic underestimation of subjects’ highest NO_2_ commuter estimates and overestimations of the lowest values in both PolluMap and ESCAPE models was found. The relationship of the NO_2_ commuter concentrations between the model pairs PolluMap-PROKAS was best fitted by a quadratic function (*R*^2^ = 0.70), and between ESCAPE-PROKAS by a cubic function (*R*^2^ = 0.77). Average differences (and SD) between time-weighted NO_2_ commuter exposure estimates of the three model pairs PolluMap-PROKAS, ESCAPE-PROKAS and PolluMap-ESCAPE within Basel-City were 0.97 (±3.12), −1.08 (±3.71) and −2.04 (±2.61) µg m^−3^, respectively (Figure 4). Differences were significantly different from 0 (tested by a Wilcoxon signed rank test).

### 3.3. Commuter NO_2_ Concentration, Exposure and Dose by Travel Mode

**Figure 5:**
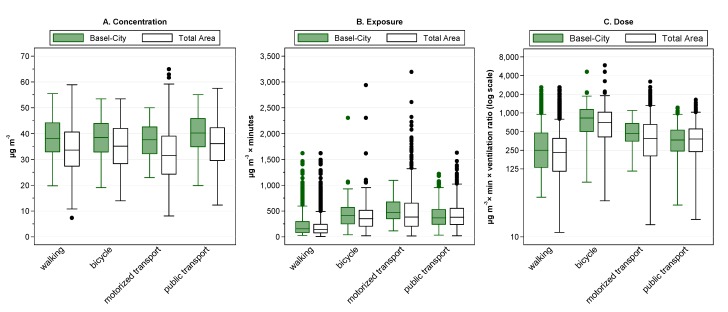
Box plots of in-traffic NO_2_ concentration (**A**); exposure (**B**); and dose (**C**) by travel mode and study area using the PolluMap model. Estimates are based on commute legs: boxes represent 25th to 75th percentile, central line the median, bars outside the box represent the most extreme values within 1.5 × the inter quartile range of the nearer quartile, and circles are outliers.

The number of legs within Basel-City (and total study area) by travel mode walking, bicycle, motorized transport and public transport were 636 (1,614), 204 (385), 58 (602), and 259 (735), respectively. Based on the commuter legs, in-transit concentration, exposure and dose are displayed by travel mode in Figure 5. Results are shown for the PolluMap model to allow comparisons between study areas. Within Basel-City, the median NO_2_ commuter concentrations estimated by the PolluMap model for walking, bicycle and motorized transport were rather similar (~38 µg m^−3^), and were slightly lower than for public transport (40 µg m^−3^). A different modal pattern emerged when considering the travel time spent in the travel modes. Highest median cumulative exposures (*i.e.*, the concentration multiplied by the duration) with the PolluMap model were obtained for motorized transport (468 µg m^−3^ × minutes) and bicycle (414 µg m^−3^ × minutes), and the lowest for walking (156 µg m^−3^ × minutes). The highest median dose was observed for bicycle commutes (829 µg m^−3^ × minutes × ventilation ratio) in the model where a two-fold increase in minute ventilation was assumed for bicycle *versus* public and motorized transport. Walking remained the mode with the smallest dose (266 µg m^−3^ × minutes × ventilation ratio), although a ventilation ratio of 1.7 relative to motorized transportation was applied. In the total study area, the modal pattern was similar to the one in Basel-City, albeit mode-specific NO_2_ estimates were generally lower.

With the higher resolution model, PROKAS, more variability in travel mode-specific commuter NO_2_ estimates was observed (data not shown). In addition, for the active transport legs—more often happening on side streets—the PROKAS model obtained 1%–2% lower estimates than the PolluMap model. In contrast, PROKAS provided 5%–6% higher estimates for passive transport legs which happen more frequently on busy roads. The percentage of the legs assigned as road class main roads within Basel-City to walking, bicycle, motorized and public transport legs was 23%, 35%, 52% and 61%, respectively (Table S2).

## 4. Discussion

The exposure to traffic-related air pollution during commute of the population living and working within Basel-City and Basel-Country was estimated using spatially and temporally resolved commuter route data, information on travel modes used and three NO_2_ air pollution models with different spatial resolutions. Within Basel-City, estimated average time-weighted population exposure was similar between all models (around 39–41 µg m^−3^). Compared to the dispersion model with the highest resolution, both the LUR model (applied to a 50 × 50 m grid) and the nation-wide dispersion model PolluMap (grid size 100 m), underestimated the concentrations on the higher end, and overestimated the values on the lower end. In the total study area, including also Basel-Country, average time-weighted commuter exposure estimated with just the PolluMap model was 34 µg m^−3^. Commuter estimates from the same model showed greater variability and covered a wider range in the total study area (12.4–52.2 µg m^−3^) than within Basel-City (range: 24.1–51.0 µg m^−3^).

Only a few studies have estimated NO_2_ in-transit exposures based on travel routes. De Nazelle *et al.* [36] extracted NO_2_ exposures from an annual dispersion model in Barcelona based on Global Positioning System (GPS) tracks from 36 working adults. The temporally adjusted in-transit exposure was twice as high as our estimates within Basel-City, illustrating both higher in-transit NO_2_ concentrations and the higher urban background NO_2_ concentration level in Barcelona (Spain). In Flanders and Brussels (Belgium), Dhondt *et al.* [19] predicted an average in-traffic population exposure of 38 µg m^−3^ over the total area using an activity-based transport model. In an exposure simulation study at census tracts level in Vancouver (BC, Canada) , annual average hourly means of NO_2_ levels were 34 µg m^−3^ on highways and arterial roads and 26 µg m^−3^ on less important roads using a dispersion model and census data [15].

To our knowledge, this is the first time that three air pollution models with different spatial scales were compared for estimating commuter exposure in the same area. We found more within-city and within-subject variability in NO_2_ concentrations with the city-specific dispersion model PROKAS than with the LUR model and the nation-wide PolluMap dispersion model. LUR models have been shown to better reflect the spatial variability of traffic-related pollutants within an urban area than conventional dispersion models [25,37] or inverse-distance weighted interpolation of monitoring data [15,23]. Compared to dispersion models, less spatially resolved input variables are required for LUR models to accurately predict within-city variability of traffic-related NO_2_ [26,37]. In our case, the NO_2_ PROKAS dispersion model performed somewhat better in our commuter exposure simulations. Beelen *et al.* [33] showed that the accuracy of LUR models to predict NO_2_ concentrations depends on the quality of the monitoring data and/or GIS variables. In particular, local traffic-intensity data have been shown to be important for achieving good model performance. The moderate model *R*^2^ of 0.67 of the LUR model used in this study is likely reflected by the limited availability of traffic input variables and possible the limited contrasts in traffic density in the City of Basel. The comparison with measurements from street sites supports this finding. However, it must be emphasized that, our LUR model was applied at a grid resolution of 50 × 50 m; therefore, the decrease in model performance may be due to both, the chosen resolution and the intrinsic limitation of the LUR model. In addition, our validation of the models with fixed-site NO_2_ measurements is not fully appropriate for the typical exposure during commute because measurements do not represent the concentrations on the traffic routes but rather home outdoor concentrations.

Comparing the two dispersion models, the model with the higher resolution showed greater variability between commuter exposures, and better agreement with measurements at street sites. The inclusion of traffic data and meteorological parameters at a more local scale and additional consideration of the building structure likely explain the higher variability, and wider range in commuter exposure estimates of the PROKAS model, and also the higher validity observed in the comparison with street sites measurements. Dispersion models have difficulties to predict within-city contrasts when interpolating meteorological data from sparse weather stations and from emission inventory data of low resolution [25]. Underestimation of NO_2_ concentration at street sites was also observed earlier in the previous version of the PolluMap model (year 2000, 200 × 200 m) [22].

Our comparison of the three models in Basel based on simulated commute exposure estimates suggests that the decision on the model to be used to estimating commuter exposure in long-term epidemiological studies depends on the aim of the study and the size and geographic diversity of the study area. For estimating commuter exposure within urban areas and examining small-scale variability between road classes, a model with a high resolution representing well the urban street environment is recommended. This seems to be especially relevant for exposure assessments within a city, where inclusion of local traffic variables of sufficient quality (hourly traffic counts, street configurations) in the model is indispensable. For larger scale longitudinal epidemiological health assessment studies, however, models with a coarser spatial resolution might be sufficient, especially when a study area is comprised of a mix of urban, suburban and rural regions. Also, higher resolution dispersion models that include detailed traffic and 3D building data, are particularly costly to develop, need adequate expertise and are often limited in spatial coverage.

Our in-transit NO_2_ estimates of long-term exposure were not validated with personal measurements. In line with the vast majority of epidemiological studies on long-term health effects of air pollution, our evaluation relies on the accuracy of the ambient models rather than personal measurements. Our objective was the estimation of exposure to air pollution during commute, using NO_2_ as the marker of traffic-related air pollution. As in the epidemiological studies, we were not interested in total personal exposure to NO_2_ *per se* as this would describe a mixture of exposure to pollution from traffic, gas cooking and other sources of combustion. Accordingly, our approach relies on the same ambient models used to derive home outdoor concentrations. Additional improvements in commuter exposure estimates may be expected when combining modelling methods with personal exposure data, as for example in hybrid models [26].

The strength of this study is the detailed data on travel behavior of a representative subset of the population. We had spatially and temporally resolved data on each leg of a commuter trip including information on travel modes used, time of day and locations where the mode of transport was changed. Our comparison of the cumulative NO_2_ commuter exposures and doses by leg shows considerable differences between travel modes and thus indicates the importance of differentiating between travel modes and related routes and travel times. Furthermore, unlike other exposure simulation studies, the estimation of commuter exposure was based on real geo-coded travel routes of a population. In this study, motorized and public transport legs comply closely with actual travel routes and are not based on assumptions. Simplified trip simulations in other studies such as the shortest route or straight line between two locations, zones or census tracts [15,16,17] may add uncertainties as drivers may prefer other routes avoiding red lights and congestions. In a short validation study with test persons (data not shown), car routes between home and work locations often did not correspond to the shortest or fastest route within the city of Basel. Thus, verifying car routes >3 km likely helps to prevent misclassification of air pollution exposure (Supplementary Section 3). However, walking and bicycle legs were also based on the shortest routing algorithm in this study. Cyclists, especially, may choose to avoid main roads and thus may have longer commuter distances. Several studies have shown that travelling by bicycle along a greener route reduces both exposures [7,38] and dose [39]. Therefore, exposure levels may be overestimated when assuming shortest routes [11]. Our comparison of the reported travel distance against routing distance, however, aimed to control for large route discrepancies (see Supplementary Section 2).

Our exposure simulation—besides potential inaccuracies of the air pollution models *per se*—had some sources of uncertainties. Comparison between travel modes are based on the spatial location of the route, distances and durations. We did not take into account travel microenvironments such as in-vehicle exposure modification due to the potential use of ventilation systems or the commuter’s position on the road. Therefore, we may have over- or underestimated in-vehicle NO_2_ concentrations. To our knowledge, there is no extensive measurement campaign of NO_2_ exposures between travel modes available, and literature on in-vehicle exposure modification of NO_2_ is very rare. Short-term measurements by Harrison *et al.* [9] in London found higher levels in buses (39 µg m^−3^) than in cars (25 µg m^−3^) or trains (16 µg m^−3^). A study by Chan and Chung [40] found significant differences in the indoor:outdoor (I/O) ratio for various ventilation modes and outdoor environments when driving in Hong Kong. On urban streets, a mean NO_2_ ratio of 0.8, 1.0 and 0.6 were reported for fresh-air intake, open windows, and air-recirculation, respectively. Ventilation characteristics of the vehicles vary by season and other vehicle characteristics. Therefore, an integration of different ventilation characteristics are expected to be small in the annual mean commuter estimates. In addition to ventilation characteristics, differences between mode-specific concentration levels and between studies vary by various factors such as meteorology, traffic parameters, and vehicle type, thus generalization from one study to another may not be appropriate [3]. A recent UFP monitoring study along a main road in Basel by Ragettli *et al.* [7] observed higher levels while driving a car or cycling compared to walking and public transportation. However, no consistent correlations between in-transit concentrations of UFP and NO_x_ have been reported [3], and therefore no modification was applied in this study. De Nazelle *et al.* [36] used ratios of BC concentrations between transportation microenvironments as a proxy for NO_2_ ratios, which explains in part higher commuter exposures found in that study. Yet another limitation of this study was the relatively small area of the study. Commutes of the Basel population to other cities within Switzerland could not be included. It must be assumed that mean commute-related NO_2_ exposure and dose would be higher when including people spending more time on their daily commutes especially when commuting on highways and in tunnels [12,21,41].

So far, epidemiological studies on long-term effects of ambient air pollution rely on home outdoor concentrations to estimate total exposure. The expansion of this approach to integrate outdoor concentrations at work or school addresses—the second most frequent location of time—is straightforward. Our approach targets at the improvement of total exposure estimates for epidemiological studies on long-term health effects through integration of the third most important time window, namely commute related exposure to ambient air pollutants. The average annual time-weighted commuter exposure estimates (34–41 µg m^−3^) in the total study area were higher than the annual mean NO_2_ concentration at the suburban background (24 µg m^−3^) but similar to the urban background station in Basel-City (30 µg m^−3^). Therefore, the contribution of commute to total NO_2_ exposure and the related effect on long-term health outcomes might be small for the majority of the population in Basel. However, for some subgroups of the population the commuter exposure could be more important, as indicated by the range of NO_2_ exposures (Table 3). Further studies may expand toward the integration of other microenvironments such as time activity patterns during leisure time.

## 5. Conclusions

We provide an approach to simulate commute routes and related exposure to traffic-related NO_2_ that can be used to improve both in-transit exposure estimates and total daily exposure estimates for epidemiological studies assessing long-term effects of air pollution on health. Information to be collected from the study population should include home and work location, travel mode, travel behavior (number of trips within a day and week, travel duration) and route (fastest *versus* shortest route, detours, and habits on avoiding main roads). The relative contribution of these commuter estimates to total daily exposure needs to be investigated and further research is needed to validate such simulations.

The decision on which air pollution model to be used depends on the aim of the study, the local situation, and on practical issues. In general, it is important to gain an understanding of the available models, and to consider the type of information and uncertainty that could emerge when using one model over another. We recommend using air pollution models which represent well the urban street network within a city when being interested in small-scale variability and differences between travel modes. Our analysis indicates that for epidemiological health assessment studies over a larger geographic scale covering rural, suburban and urban areas, however, models with a coarser spatial resolution are likely adequate, but need to be formally evaluated.

## Supplementary Files

Supplementary File 1 Supplementary Information (PDF, 236 KB)

Supplementary File 2 Supplementary Information (PDF, 373 KB)
