# Supplementary material for: Simulation of Population-Based Commuter Exposure to NO2 Using Different Air Pollution Models
Source: Int J Environ Res Public Health. 2014 May 12;11(5):5049–68. doi: 10.3390/ijerph110505049 (PMC4053908; doi:10.3390/ijerph110505049)
Supplement: Supplementary File 1 — Supplementary Information (PDF, 236 KB) [file ijerph-11-05049-s001.pdf]

Supplementary Information

Simulation of Population-Based Commuter Exposure to NO_2 Using Different Air Pollution Models

1. Validation of GIS Model

In order to validate the performance of the GIS commuter model used to simulate the walking and bicycle legs in this project, a small validation study was carried out. Information on commuter route and behavior was collected from 36 subjects of the Swiss Tropical and Public Health Institute. First, participants were asked to estimate the duration and distance of their commuter route between home and work locations, as well as to provide information on their commuter mode and route characteristics. Home and work addresses were also collected. Second, participants showed their exact route in Google Map (true distance). Finally, participants' commuter routes between home and work locations were simulated by means of the GIS model developed for this study. Figure S1 shows the comparison between the routing distance (from the GIS model) and the true distance (measured in Googe Map) and reported distance, respectively, for walking and cycling trips. Results are shown for subjects that only walk or cycle to work (*i.e.*, do not combine various travel modes). There is a high agreement between the true distance and the simulated distance (R^2~1.0). Comparisons between the reported distance and the modeled distance are similar to the comparisons with the Microcensus data (Figure S2).

Figure S1. Comparison of simulated routing distance against distance measured in Google map as well as reported distance in a pilot study in Basel.

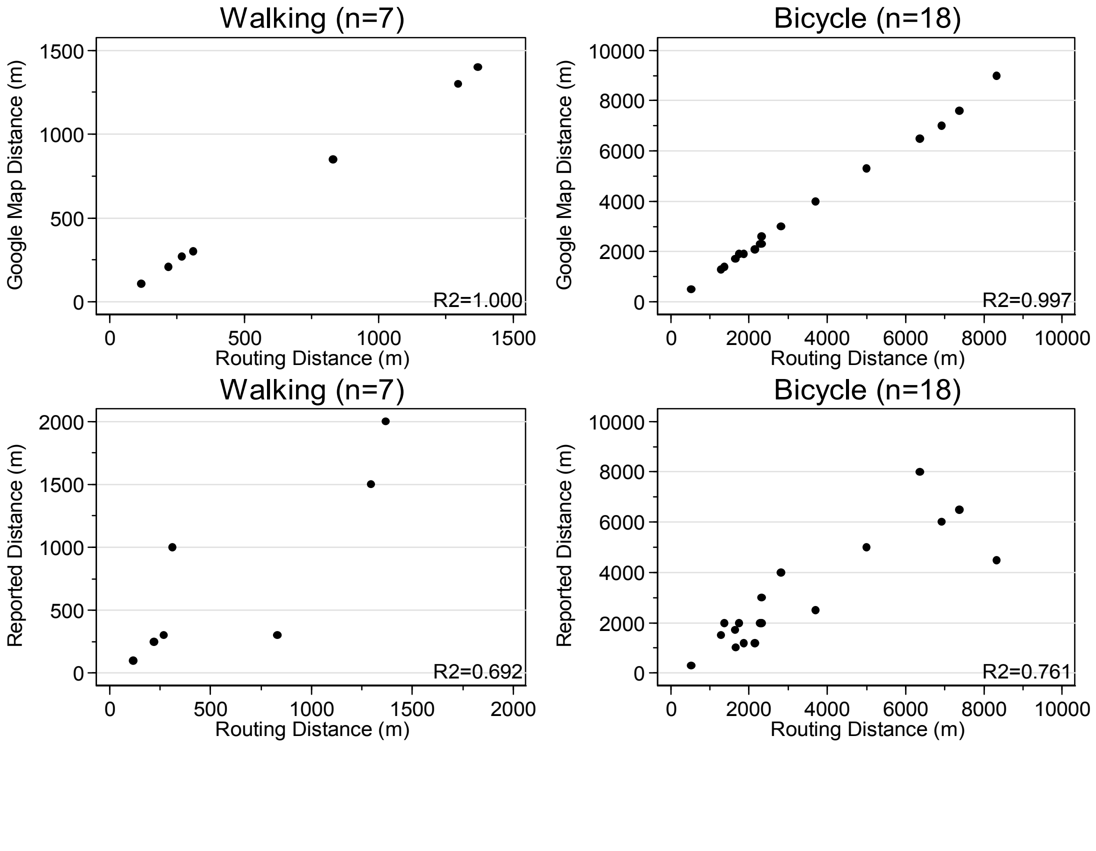

2. Comparison between the Routing Distance and Reported Distance Provided by the Microcensus Data 2010

Routing distance and reported distance (estimated by the subjects during the interview) were compared (Figure S2) to avoid detours and ensure the plausibility of the legs. Outliers were checked manually and excluded from the analysis if they did not make sense.

Figure S2. Reported *versus* routing distance. The reported distance was estimated by the subject during the interview.

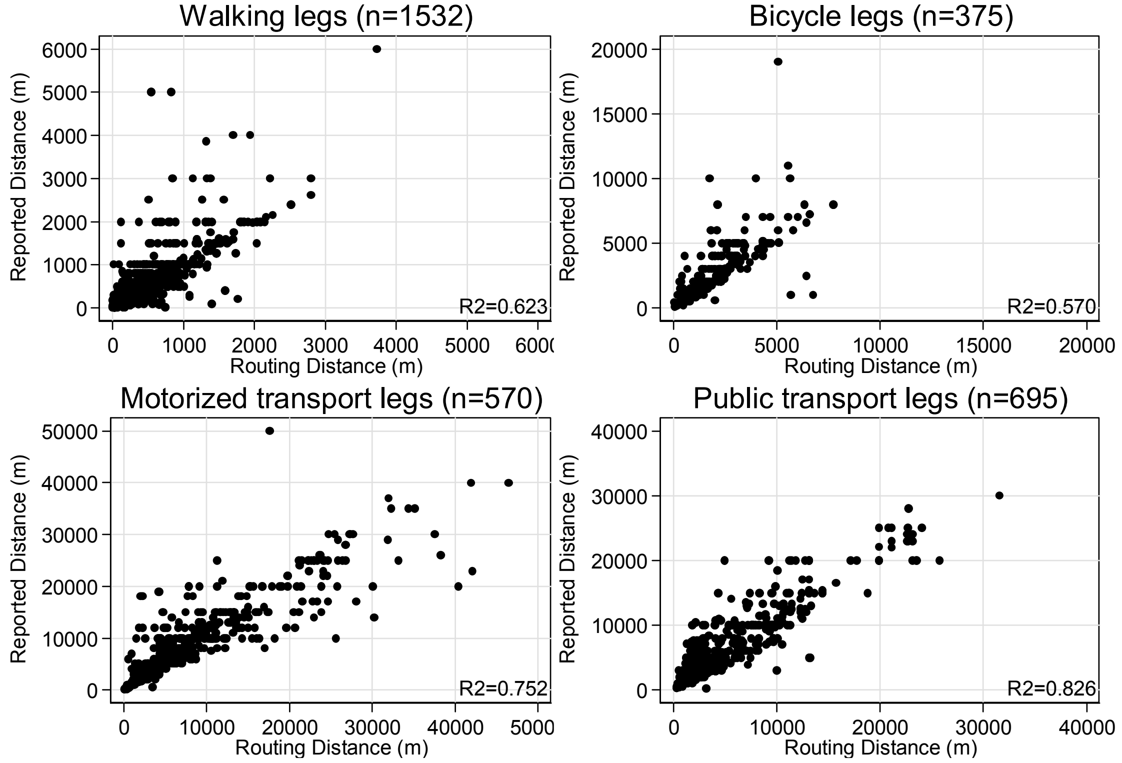

3. Comparison of Commuter NO_2 Exposure Estimates along Verified and Fastest Car Trips >3km Between Home and Work/School Locations.

We assessed the difference in commute exposure estimates that occur when modeling NO_2 exposure for car trips along the fastest as opposed to the verified route between home and work/school. A subsample of 91 subjects was selected that only commuted by car between home and work/school and had commuter trips longer than 3 km. The verified car trips correspond to the route that was reported during telephone interviews. The fastest route was subsequently simulated based on the same road network (TeleAtlas MultiNet). The PolluMap model was used to estimate commuter exposure to NO_2. Comparing the time-weighted subjects' NO_2 commuter exposure estimates of the two approaches resulted in an R-square of 0.5 (Figure S3). Overall, the mean (±standard deviation) of exposure estimates along the fastest routes (32.2 ± 11.2 µg m^{-3}) was similar to the ones along the verified routes (33.3 ± 8.7 µg m^{-3}). Absolute differences between the corresponding estimates had mean of 5.5 (±6.1) µg m^{-3}.

Figure S3. Subjects' NO_2 commuter exposure (estimated using the PolluMap model) along verified versus fastest car routes between home and work/school locations. Only subjects who commuted by car and had car trips >3 km were included.

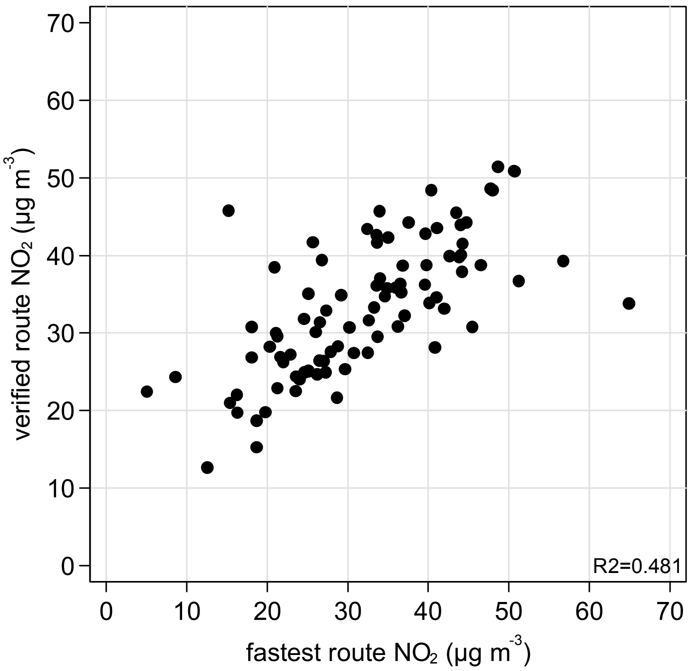

4. ESCAPE Basel NO_2 Model (12 April 2012) (Mostly published in Beelen *et al.* 2013, Atmos Env 72(2013) 10–23)

Table S1. Basel NO_2 ESCAPE model: (**a**) describes the model (with VIF—Variable Inflation Factor); (**b**) its performance (with Maximum Cook's distance); (**c**) its leave-one-out cross-validation; and (**d**) its Moran's I.

		**(a) The Model**			
	Estimate	Std. Error	t value	Pr(>|t|)	VIF
(Intercept)	5.435E+01	3.306E+01	1.64	0.109	
INTMAJORINVDIST	1.227E−02	2.244E−03	5.47	0.000	1.082
RES5000_500	5.330E−07	2.452E−07	2.17	0.037	1.482
SQRALT	−3.956E+00	1.523E+00	−2.60	0.014	1.379
RES500	1.878E−05	1.063E−05	1.77	0.086	1.181
		**(b) Performance**			
	R2	Adj_R2	RMSE	MaxCooksD	
Basel ESCAPE model	0.67	0.63	4.48	0.16	
		**(c) Cross Validation**			
	CV_R2	CV_Adj_R2	CV_RMSE		
Basel ESCAPE model	0.58	0.57	4.83		
(**d**) Moran's I					
	observed	expected	sd	*p* value	
Moran's I	−0.05	−0.03	0.03	0.45	

Predictor Definitions:

INTMAJORINVDIST:	Product of traffic intensity on nearest major road (INTMAJOR) and inverse of distance to the nearest major road (INVDIST). [Veh/day/m]
RES5000_500:	Residential land in a donut with outer radius of 5,000m and inner radius of 500 m. [m^2]
SQRALT:	Square root of altitude. [$m^{1/2}$]
RES500:	Residential land in a circle of radius 500 m. [m^2]

5. Temporal Adjustment

We calculated temporal adjustment factors for each hour of the day separately for main roads and side streets to consider the diurnal pattern of NO_2 levels and differences in hourly traffic volume and composition of vehicles (*i.e.*, with separate counts for personal cars and trucks). Ratios were computed between the annual weekday hourly means and the annual mean concentration measured at the monitoring stations (see Figure S4). While the ratios at the side road were peaked during rush hours, those at the street site were highest during working hours (9–11am; 3–5pm), most likely due to increased truck traffic. Ratios were applied to each commuter leg concentration based on the road classification and start hour of the leg. Table S2 shows the percentage of legs defined as main and side roads separately for study area. Summary statistics of the ratios are provided in Table S3.

Figure S4. Number of commuter legs per hour of the day and annual weekday hourly NO_2 means (±standard deviation) at the two fixed stations (main road and side road) used to compute temporal adjustment ratios. The straight lines represent the annual average NO_2 measured at the two fixed stations.

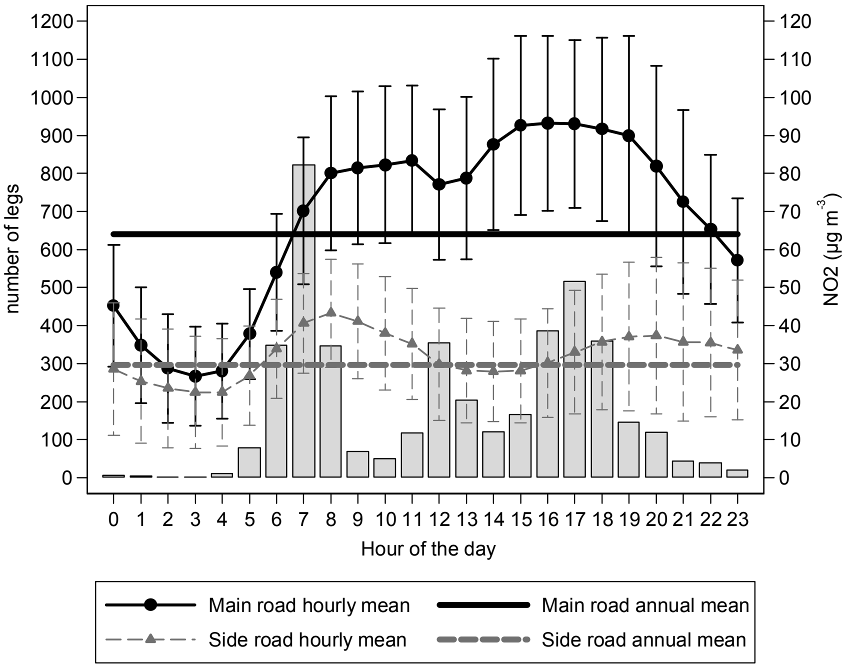

Table S2. Street class applied to legs by travel mode and study area.

Travel Mode	Study Area	N (Legs)	Main Road (%)	Side Road (%)
walking	Basel-City	966	22.6	77.4
	Total area	2583	23.3	76.7
bicycle	Basel-City	204	34.8	65.2
	Total area	385	29.9	70.1
motorized transport	Basel-City	58	51.7	48.3
	Total area	602	79.7	20.3
public transport	Basel-City	259	60.6	39.4
	Total area	733	56.9	43.1
other	Basel-City	18	0.0	100.0
	Total area	37	0.0	100.0

Table S3. Summary of ratios applied to legs and waiting points by study area and road class.

Study Area	Variable	n (Legs)	Mean	(sd)	Median	Min	Max
Basel-City	main road	476	1.25	(0.18)	1.25	0.59	1.45
	side road	1029	1.17	(0.18)	1.14	0.76	1.46
Total Area	main road	1615	1.24	(0.22)	1.25	0.44	1.45
	side road	2725	1.18	(0.17)	1.14	0.76	1.46

6. Comparison of the Three NO_2 Models with Measurements

We compared the three models—PROKAS, ESCAPE and PolluMap—to NO_2 measurements from a total of 31 sites within Basel-City from the Swiss study on Air Pollution and Lung and Heart Diseases in Adults (SAPALDIA) (Table S4, Figure S5). These measurements were conducted outside subjects' homes in three biweekly integrated sampling campaigns in 2011 using Passam passive diffusion samplers (Passam AG, Schellenstrasse, Männedorf, Switzerland). To keep the evaluation spatially comparable, we compared the average NO_2 concentrations of each site to the value estimated for the grid value of the three models. Thus, the data do not reflect a proper validation of the models.

Table S4. Comparison between modeled and measured [a] NO_2 (in µg m^{-3})

Model	Street Sites (*n* = 18)			Urban Background Site (*n* = 11)			All Sites [c] (*n* = 31)		
	R^2	Bias [b]		R^2	Bias [b]		R^2	Bias [b]	
		Mean	(sd)		Mean	(sd)		Mean	(sd)
PROKAS	0.44	−2.1	(4.4)	0.50	−1.6	(3.6)	0.58	−1.9	(4.0)
ESCAPE	0.15	−3.6	(5.4)	0.34	−4.4	(4.4)	0.41	−3.7	(4.8)
PolluMap	0.17	−4.1	(5.2)	0.67	−4.7	(3.1)	0.46	−4.3	(4.4)

Note: [a] Measurements were conducted during three seasons (two-week samples) in 2011 as part of the Swiss SAPALDIA study (Basel-City only); [b] The bias is calculated as the difference (predicted-measured) NO_2, shown as mean and standard deviation (sd); [c] Two sites are classified as regional background sites.

Figure S5. Comparison of NO_2 models with measurements of 31 sites from the 2011 SAPALDIA study.

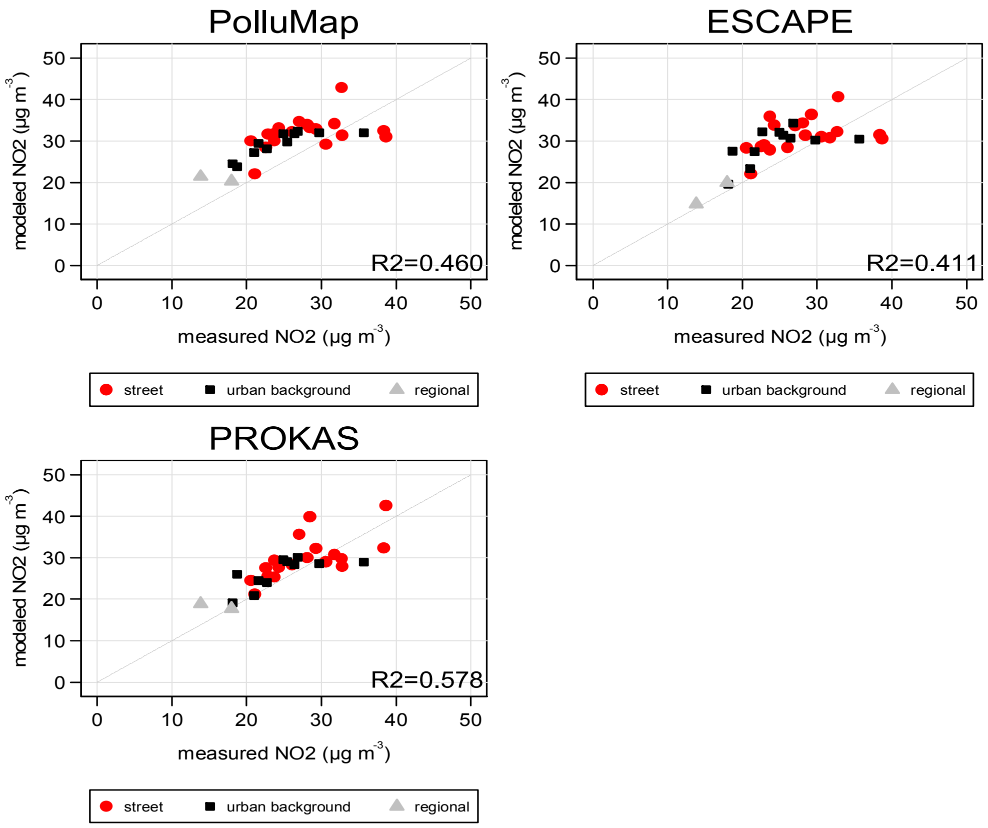

7. Study Population

Table S5. Characteristics of the study population.

		Basel-City	Total Area
subjects	n	258	736
female	%	59.3	50.3
age	mean (sd)	35.0 (17.0)	36.7 (17.6)
work fulltime (≥90%)	%	51.2	53.8
work ≥50%–89%	%	16.3	16.4
work <50%	%	7.0	7.6
in education	%	7.4	5.7
<15 years old	%	18.2	16.4
subjects with 2 commuter trips/day [a]	%	84.1	84.4

Note: [a] The rest of the subjects had four trips per day.

8. Spearman Correlation Coefficients between NO_2 Models

Table S6. Spearman correlation coefficients of daily NO_2 commuter estimates between the three air pollution models in the study area Basel-City.

Model Comparison				
	n	PROKAS-ESCAPE	PROKAS-PolluMap	ESCAPE-PolluMap
concentration (legs)	1175	0.89	0.85	0.91
commuter concentration [a] (subject)	258	0.87	0.81	0.86
commuter exposure [b] (subject)	258	0.99	0.99	0.99
commuter dose (subject)	258	0.99	0.99	0.99

Note: [a] Sum of leg concentrations divided by the number of legs; [b] Concentrations multiplied by durations, includes waiting time between legs. All correlations are significant ($p < 0.05$).

© 2014 by the authors; licensee MDPI, Basel, Switzerland. This article is an open access article distributed under the terms and conditions of the Creative Commons Attribution license (http://creativecommons.org/licenses/by/3.0/).
